# Air Tubercles from the Living Subject

**Published:** 1858

**Authors:** 


### Air Tubercles from the Living Subject.

When are we to understand that a patient with pulmonary disease is past recovery? What beacon can we invoke to give the sign of certain death? or what answer must we receive to a prying investigation of a case, to believe in a recovery? In other words, how far may a patient die and yet recover?

Dr. Cartwright, of New Orleans, when adulating the Sugar-house treatment of Consumption, remarked, that "it was, of course, not expected of that treatment, to permanently benefit invalids with cavities in their lungs"—thereby indicating his lack of faith in the recovery of any case, where a cavity had formed. Is not a cavernous lung a fatal lesion, in the opinion held among medical men generally, notwithstanding medical literature tells us of recoveries after their formation? Most phthisical subjects die in defiance of the best-directed medication, but an occasional case will recover in some surprising manner, after a majority of the well-defined fatal symptoms have become conspicuous. Such cases give room to dispute the accuracy of a diagnosis which supposes the existence of cavities, or infers large destructive inroads into the pulmonary structure. They also pronounce a commentary, widely at variance with the prevalent opinion, touching the final issue. They enlarge the boundary of the patients' hopes, and introduce them to the contemplation of extravagent possibilities.

A rare case wherein Nature, who does everything lawfully, though it be irregular, is the following. With marvelous temerity she tried the experiment, as if to illustrate how far a consumptive may die and yet recover.

The first of February, 1857, Mr. Hubbard was visited with an attack of lung disease, bearing the general marks of pneumonia. He has for many years been subject to these attacks. They most frequently occur in the Spring, but often in the Winter. Pain in the chest, frequent and bloody expectoration usually attend them.

When called to prescribe, he was found laboring under much prostration of strength, absolute loss of appetite, thirst, hot and dry skin, alternated with free perspiration; had active cough, and expectorated tenacious mucous, stained with blood; pulse numbered one hundred and twenty.

A pectoral blister was applied, and ten grain doses of camphorated Dover powder and quinine were left, to be given every three, six or twelve hours, at discretion; vinous spirits to be taken three or four times a day. The pain in the chest, and the violence of the cough were soon allayed, but he appeared to fail from day to day. His strength failed, and he was obliged to keep his bed. His breathing became oppressed, short and difficult. The best knowledge the auscultating ear could furnish of the progress the hidden mischief had made, was a cavernous left lung. An unfavorable result was expected. Cough continued, and the expectoration, touched and streaked with blood, was wearing him away. It was tenacious and stringy, often requiring the aid of the fingers to effect its liberation from the mouth. Once his fingers siezed a fibrous, and so well defined a cord, which lingered in the mouth, that he was curious to have it examined. Freed from mucous it unfolded itself in water, a beautiful, clear, white, pulmonary air tube, about two inches in length.

He continued to expectorate these fragments of disintegrated structure for many days. A dozen or more specimens I have preserved in my cabinet. How long, prior to this discovery, they were thrown off we have no means of knowing, since the outward appearance of them in the sputa so resembled bloody mucous, as to pass collapsed and unobserved.

Meantime his respiration became more difficult, he required his shoulders raised and the windows opened to obviate defective aeration of blood. His pulse rose to one hundred and forty and one hundred and fifty to the minute. His countenance was sunken, anxious and staring; his skin, livid and bathed in perspiration. He spoke his accents between times of gasping for breath.

These extreme evidences of immediate dissolution would last a few hours, and then subside, to return the next or succeeding day. Thus he continued for about a week, still taking, occasionally, Dover powder and quinine, but spirits constantly.

At length the symptoms abated their violence. He began to breath more freely and rest better and cough less. He felt like eating something, and then began to mend about as steadily as he had before declined. Though by no means stout, yet he has recovered his usual degree of health, and is out at work, and I have particular portions of his lungs in a bottle.

